# A comparison of Gaussian processes and polynomial chaos emulators in the context of haemodynamic pulse–wave propagation modelling

**DOI:** 10.1098/rsta.2024.0222

**Published:** 2025-03-13

**Authors:** L. Mihaela Paun, Mitchel J. Colebank, Dirk Husmeier

**Affiliations:** ^1^ School of Mathematics and Statistics, University of Glasgow, Glasgow, UK; ^2^ School of Mathematical Sciences, University of Southampton, Southampton, UK; ^3^ Department Biomedical Engineering, University of California, Irvine, CA, USA; ^4^ Department of Mathematics, University of South Carolina, Columbia, SC, USA

**Keywords:** emulation, Gaussian processes, polynomial chaos expansions, sensitivity analysis, parameter estimation, pulmonary pulse–wave propagation modelling

## Abstract

Computational modelling of the cardiovascular system is a promising future direction for patient-specific healthcare. However, the computational cost of these simulators is a bottleneck for their practical use in clinic for real-time *digital twins*. Emulation can overcome this, yet an extensive investigation into cardiovascular emulators is warranted. In this study, we emulate two one-dimensional haemodynamics models of the pulmonary circulation and compare two common emulation strategies: Gaussian processes (GPs) and polynomial chaos expansions (PCEs). We start by reducing the parameter space of the models through global sensitivity analysis, and then compare both emulation strategies using a multivariate, time-series output quantity of interest and a reduced representation using principal component analysis. We compare the emulators in both forward emulation on test data, as well as in their ability to infer parameters in the inverse problem. Our results indicate that GPs slightly outperform PCEs consistently across every comparison, and that a similar performance is obtained for the emulators of the time-dependent output and reduced output.

This article is part of the theme issue ‘Uncertainty quantification for healthcare and biological systems (Part 1)’.

## 1. Introduction

Computational modelling and simulation is a useful tool for understanding complex physical processes. These tools have been developed and adapted to handle cardiovascular physiology with a hope of developing a cardiovascular ‘digital twin’ [1]. Models that simulate *in vivo* haemodynamics are complex, and should account for both the structure and function of the vascular system. A notable example is the image-based simulation platform Heartflow©, which uses a combination of image analysis, computational haemodynamics and machine learning to predict coronary artery disease risk factors [2]. The clinical success of these techniques is due in part to reliable, robust simulation techniques (i.e. computational fluid dynamics), but also because of their computational speed-up using *emulation*.

Several studies in the literature compare emulation strategies [3–6]. For example, the study by Laloy *et al*. [3] compared Gaussian processes (GPs), polynomial chaos expansions (PCEs) and deep learning neural network (NN) emulation of reactive transport models. The authors compared output predictive accuracy, global sensitivity analysis results and probabilistic model calibration, and found that the GP was the most robust, being the only method that performed well across all considered tasks. The study by Pratola *et al*. [4] compared a Bayesian Additive Regression Tree model of a multivariate output response with GP emulators using a principal component analysis (PCA) reduction of the output space with respect to model calibration in a CO_2_ emission model. The study found no significant difference between the two emulation approaches with respect to most model parameters inferred, with the former method having the advantage of not requiring a dimension-reduction step. Other studies [7–9] have implemented PCE in combination with proper orthogonal decomposition (POD), similar to PCA, which relies on the leading eigenpairs for dynamic outputs. Though POD can be used to reduce the computational cost of expensive cardiovascular simulation [8], it is rarely used for the purpose of solving inverse problems. In cardiovascular applications, both GPs and PCEs are commonly used [10–13], but are rarely compared, and the trade-offs in how these emulation strategies perform in forward emulation or in solving inverse problems are unknown.

To address this gap in the literature, our study provides a detailed comparison of GPs and PCEs for the emulation of pulmonary blood pressure in a pulse–wave propagation model. We compare emulation strategies using two different representations of the training data: a multivariate output (time series) generated by a forward simulation using the simulator, and training data described by a PCA representation of the multivariate output. We begin by conducting a global, variance-based sensitivity analysis to establish an influential set of model parameters for two distinct sets of boundary conditions. Besides using the GPs and PCEs for direct emulation, we also investigate their use in the context of parameter estimation (or model calibration). Our study shows that GPs consistently, but slightly, outperform PCEs across all error metrics, training data representations and simulator boundary conditions.

## 2. Haemodynamics model

The haemodynamics model is similar to our previous studies [14–16], and simulates arterial pulse–wave propagation in both time, t (s), and axial space, x, throughout a bifurcating, murine network of 21 blood vessels. Pulmonary arterial blood pressure (p(x,t), mmHg), area (A(x,t), cm${,}^{2}$) and flow (q(x,t), ml s^–1^) are predicted using a system of nonlinear, hyperbolic, partial differential equations. The system includes a mass conservation and momentum balance equation:


(2.1) $$\begin{array}{lcr} & \frac{\partial A}{\partial t}+\frac{\partial q}{\partial x}=0,\;\;\;\;\frac{\partial q}{\partial t}+\left(\frac{\gamma +2}{\gamma +1}\right)\frac{\partial }{\partial x}\left(\frac{q^{2}}{A}\right)+\frac{A}{\rho }\frac{\partial p}{\partial x}=-\frac{2\pi \mu (\gamma +2)}{\rho }\frac{q}{A}, & \end{array}$$

respectively. We assume the blood density is ρ=1.055 (g ml^–1^), that the blood viscosity is μ=0.049 (cm${,}^{2}$ s^–1^) [14] and that the blood vessel velocity profile is blunt, with the power coefficient γ=9 [17]. We assume a linear pressure–area relationship [16], where the blood vessel material properties, $Eh/r_{0}$, incorporate the Young’s modulus, E (mmHg), the wall thickness, h (cm) and the reference radius, $r_{0}$ (cm). As in previous studies [15,18], we assume that the term $Eh/r_{0}$ increases exponentially with small vessel radii, and use the relationship $Eh/r_{0}=k_{1}\exp\;\left(-k_{2}r_{0}\right)+k_{3}$, where $k_{1}$ (mmHg) and $k_{2}$ (cm${,}^{-1}$) control the exponential increase and $k_{3}$ (mmHg) is a baseline measure of the material properties. A measured main pulmonary artery flow is used as the inflow boundary condition. We enforce flow conservation and pressure continuity at vessel junctions (see Section 1 in the electronic supplementary material, for more details).

*Terminal Boundary Conditions*: We consider two distinct boundary conditions at the end of the 21-vessel network, which are linked to 11 terminal vessels. The first is the Windkessel (WK) model [19], which is an electrical circuit with a proximal resistor in series with a distal resistor and capacitor (representing compliance) in parallel. As described elsewhere [14,20] and in the electronic supplementary material, Section 1.1, we infer global scaling factors that adjust each proximal resistance, distal resistance and total compliance by $r_{p},r_{d}$ and $c_{T}$, respectively. Hence, the six parameters for sensitivity analyses are $\theta_{\text{WK}}=\left\{k_{1},k_{2},k_{3},r_{\text{p}},r_{\text{d}},c_{\text{T}}\right\}$.

The second boundary condition is the structured tree (ST) model [18]. The ST assumes that the vasculature attached to the image-based domain is an asymmetric, bifurcating tree, geometrically described by four parameters: a large daughter radii scaling factor, α, a small daughter radii scaling factor, β, a length-to-radius ratio, $\ell_{rr}$, and a minimum radius for the ST, $r_{\text{min}}$ (cm) (see the electronic supplementary material, Section 1.2, for more details). The ST has its own set of stiffness parameters, as well as the four structure tree parameters, giving $\theta_{\text{ST}}=\left\{k_{1},k_{2},k_{3},k_{1}^{\text{SA}},k_{2}^{\text{SA}},k_{3}^{\text{SA}},\alpha ,\beta ,\ell_{\text{rr}},r_{\text{min}}\right\}$.

## 3. Data

*Training data for forward emulation*: Both GPs and PCEs use a common, fixed design. We evaluate the simulator f(⋅) at the biophysical parameters θ using n design points $\Theta =(\theta_{1},\ldots ,\theta_{n})$, sampled from a space filling, Sobol sequence [21]. We consider two choices for the design size: n=100 and n=1000. The model output, f(θ,t), is a multivariate time series of the main pulmonary arterial blood pressure composed of m=32 points, that is, $f(\theta ,t)=(f(\theta ,t_{1}),\ldots ,f(\theta ,t_{m}){)}^{T}$, where the superscript T indicates transposition. We combine the model outputs into an m×n training dataset:


(3.1) $$\text{F}(\Theta ,t)=(f(\theta_{1},\text{t}),\ldots ,f(\theta_{n},\text{t})).$$

*Validation data for hyperparameter selection*: To select optimal hyperparameters for GPs (kernel type and jitter value) and PCEs (polynomial order), we continue the Sobol sequence and generate $n_{\text{valid}}=100$ simulator outputs, ${\text{F}}_{\text{valid}}=\left(f(\theta_{1}^{\text{valid}},\text{t}),\ldots ,f(\theta_{n_{\text{valid}}}^{\text{valid}},\text{t})\right)$, corresponding to parameter vectors $\Theta^{\text{valid}}=(\theta_{1}^{\text{valid}},\ldots ,\theta_{n_{\text{valid}}}^{\text{valid}})$. Validation data help avoid overfitting and bias, as well as over-optimistic results, which could be obtained if the same test data were used for hyperparameter selection. While overfitting is not due to noise (since we use noise-free data), it may be because of an imperfect space-filling design due to a clustering of points in certain regions of the parameter configuration space (see fig. 7.12 in [22] for details).

*Testing data*: We further continue the Sobol sequence and generate $n_{\text{test}}=100$ simulator outputs, ${\text{F}}_{\text{test}}=\left(f(\theta_{1}^{\text{test}},\text{t}),\ldots ,f(\theta_{n_{\text{test}}}^{\text{test}},\text{t})\right)$, corresponding to parameter vectors $\Theta^{\text{test}}=(\theta_{1}^{\text{test}},\ldots ,\theta_{n_{\text{test}}}^{\text{test}})$ for out-of-sample emulator testing evaluation. For inference, we use the same testing datasets, ${\text{F}}_{\text{test}}$, which are noise-free, allowing us to explicitly quantify the emulation error, which would otherwise be difficult to disentangle from signal noise in the case of real data.

## 4. Emulation strategies

**Figure 1.**
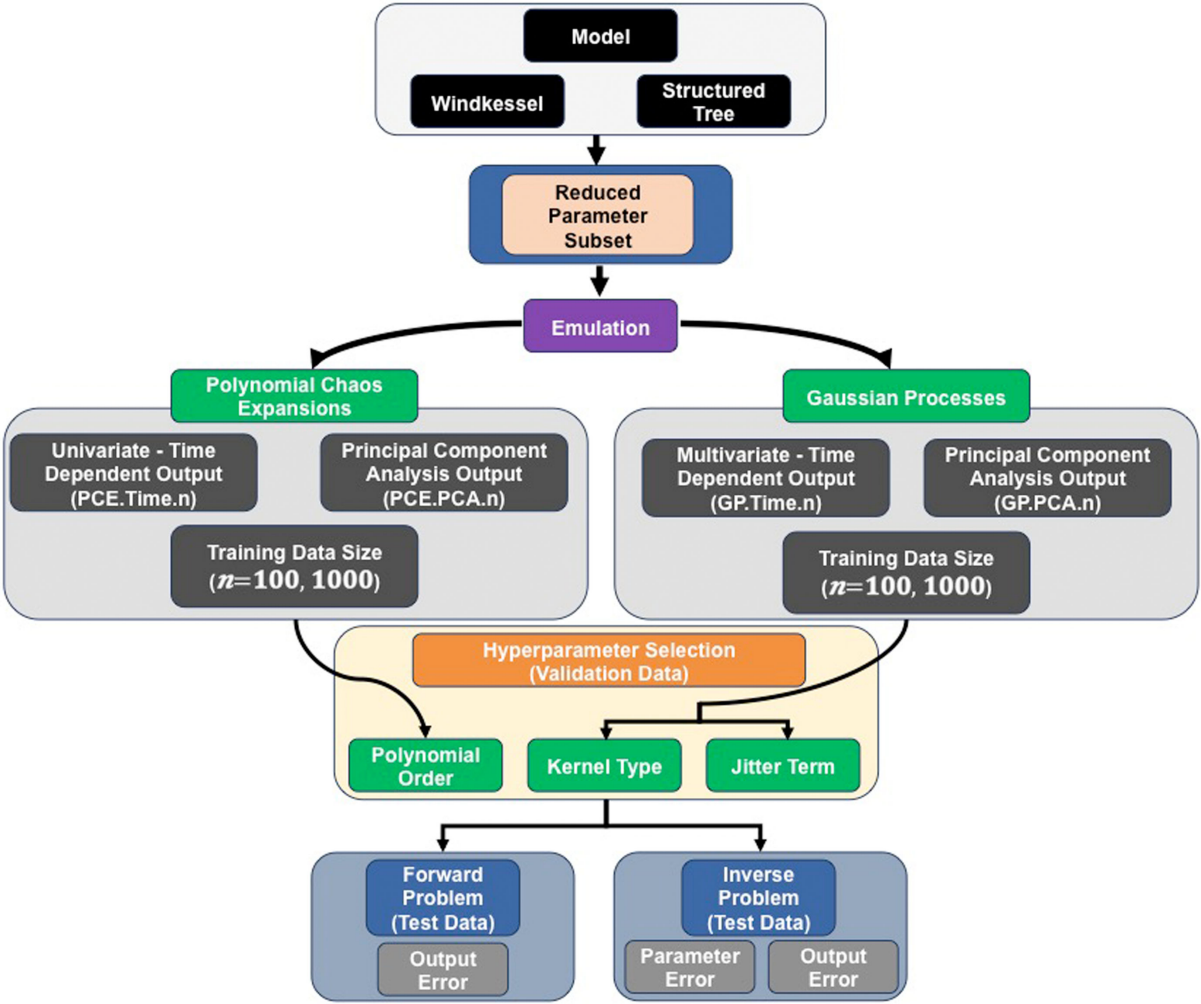
Workflow for the study. The one-dimensional model, with either WK or ST boundary conditions, is subjected to a variance-based sensitivity analysis and parameter subset reduction. Emulators (Gaussian processes (GPs) or polynomial chaos expansions (PCEs)) are then trained on the two models using n=100 or n=1000 training sets, which include either an m=32 time-series output or reduced PCA data representation (q=5 principal components). We carry out a validation study to select the optimal hyperparameters (kernel type and jitter term for the GPs; polynomial order for the PCEs). Finally, we assess the emulators with the optimal hyperparameters on test data based on forward emulation accuracy, and on parameter and output error from solving the inverse problem.

We consider emulation strategies based on GPs and PCEs, starting with a common and fixed design, defined in §3. We use two different model output representations, as described below, and summarized in figure 1.

(I) *Emulator in simulator output space*: We train the emulators for the multivariate simulator output:


(4.1) $$f(\theta ,t)=(f(\theta ,t_{1}),...,f(\theta ,t_{m})).$$

A GP for the multivariate output, f(θ,t) in (4.1) is constructed by introducing time as an additional input besides θ, and assuming separability in the joint covariance function of parameters and time. This leads to a GP model with a Kronecker product structure over the input parameters, that captures the correlation between time points besides the correlation between biophysical parameters [23]. We denote this approach as GP.time.

We also create a PCE emulator for the multivariate output using independent PCE coefficients for each output point, $f(\theta ,t_{i}),i=1\ldots m$ [24,25], and denote this by PCE.time. The PCE polynomials are the same across the individual time points while the coefficients of the polynomials are unique for each polynomial and each time point. This methodology does not explicitly account for the coefficient correlations, that is, the relationships between coefficients at $t_{i},t_{j},i\neq j$, nor does it consider more intrusive approaches to time-dependent PCE [26]. However, it is a common approach in applications with multivariate or vector responses [24,25] and is readily available in open-source software (e.g. UQlab [27]). The coefficients are determined through a regression approach, as discussed more later.

(II) *Emulator in PCA-reduced space*: We build an emulator on a reduced representation of the multivariate simulator output in (4.1) using PCA [28]. PCA retains most of the information from the original output, and captures correlations between outputs at different time points. The simulator output is decomposed into a linear combination of basis vectors $\gamma_{j}$:


(4.2) $$f(\theta ,\text{t})=\mu (\text{t})+\sum_{j=1}^{q}c_{j}(\theta ,\text{t})\gamma_{j}+\epsilon (\theta ,\text{t}),$$

where q<<m, μ(t) is the mean of the training simulator runs F in (3.1); basis $\Gamma_{q}=(\gamma_{1},\ldots ,\gamma_{q})$ comes from the singular value decomposition of the covariance matrix $\frac{1}{n-1}\left(\left(\text{F}(\Theta ,t)-\mu (\text{t}){)}^{T}(\text{F}(\Theta ,t)-\mu (\text{t})\right)\right)$; $c(\theta ,t)=(c_{1}(\theta ,\text{t}),\ldots ,c_{q}(\theta ,\text{t}))$ are the principal component scores and ϵ(θ,t) is the part of f(θ,t) unexplained by the basis term.

We perform PCA based on 1900 training points, that is, n=1900 in (3.1), and select five PCs, that is, q=5 in (4.2), which ensures that more than 99% of the variability in f(θ,t) is explained. Independent GP or PCE emulators are subsequently fitted to the principal component scores $c_{j}(\theta ,t),j=1\ldots q$. These approaches are denoted by GP.PCA and PCE.PCA, respectively. Below we give a brief overview about GPs and PCEs. Further details can be found in Sections 2, 3 and 5 of the electronic supplementary material.

### (a) Gaussian processes

GPs [29–32] originate from the Kriging methodology in geostatistics [33], and have become one of the most widely used approaches for emulation. This is due to their flexibility (a form of Bayesian non-parametric regression) and their ability to perform exact interpolation at training points. GPs also give a measure of uncertainty at test points due to the distributional assumptions.

*GPs with Principal Component Analysis*—We fit independent GP emulators for each principal component score:


(4.3) $$c_{j}(\Theta )|\eta ∼\text{GP}(0,K|\eta ),\;j=1,\ldots ,q,$$

where for simplicity of notation we have dropped the fixed index t from $c_{j}(\cdot )$. Here, we have assumed zero mean (after data standardization to zero mean), and $K=[k(\theta_{l},\theta_{p}){]}_{l,p=1}^{n}$ is a covariance matrix, with the kernel k(⋅|η) being a function of hyperparameters η, which are optimized through maximum likelihood. The kernel gives the smoothness and variability of the function $c_{j}(\cdot )$. We explore the following kernels: squared exponential, Matèrn ν (with ν=3/2 and 5/2) and neural network (see Chapter 4 in [29] for details), and include an automatic relevance determination option to allow each individual parameter in θ have its own length scale. Though principal component scores are deterministic, we add a low-variance noise (jitter, also known as nugget [29,34,35]), $\sigma^{2}$, to the diagonal of the covariance matrix, K, for numerical stability during GP training and evaluation. We consider several values for the jitter, that is, $\sigma^{2}=\left\{10^{-4},10^{-5},10^{-6},10^{-7},10^{-8},10^{-9}\right\}$.

GP-predicted values for PCA scores at unseen parameter vectors $\tilde{\theta }$ given training data D, $c_{j}(\tilde{\theta })|D$ are obtained using the GP predictive mean^1^ (see equation (15) in the electronic supplementary material), and are inserted in (4.2) to reconstruct the multivariate signal, $\tilde{f}=M(\tilde{\theta },t)$.

*Multioutput GPs for time series:* Rather than implement an onerous multi-output GP for the multivariate output in (4.1), we instead introduce time as an additional input to the GP besides θ [23], and the 1−1 simulator output function becomes $f(\theta ,t_{i})$, which defines a *univariate* output, and on which we place a GP, as follows:


(4.4) $$f(\Theta_{\theta ,t})|\tilde{\eta }∼\text{GP}(0,\tilde{K}|\tilde{\eta }),$$

where $\Theta_{\theta ,t}=\cup_{i=1}^{n}(\cup_{j=1}^{m}(\theta_{i},t_{j}))$ denotes the matrix of training points for physiological parameters θ and m accompanying indexing training time points (here m=32). The dataset $\Theta_{\theta ,t}$ has size n⋅m, as opposed to n of the original dataset, Θ. To avoid inverting a possibly very large matrix $\tilde{K}$, separability in the covariance function can be imposed between inputs θ and t, $k((t_{i},\theta_{i}),(t_{j},\theta_{j}))=k_{t}(t_{i},t_{j})k_{\theta }(\theta_{i},\theta_{j})$, which allows representing the full covariance matrix as the Kronecker product between two smaller matrices, $\tilde{K}(\Theta_{\theta ,t},\Theta_{\theta ,t})=K_{t}(t,t)⊗K_{\theta }(\Theta ,\Theta )$. Computational time reductions may be achieved by making use of special properties of Kronecker products, see details in Section 3 of the electronic supplementary material. The GP kernels for θ are the same as those explored for the GP.PCA approach, and for the time input we consider the periodic and Matèrn ν (with ν=3/2 and 5/2) kernels. Similar to the GP.PCA approach, we add the same jitter values to the diagonal of the covariance matrices, $K_{t}(t,t)$ and $K_{\theta }(\Theta ,\Theta )$.

Predictions for the multivariate signal $\tilde{f}=M(\tilde{\theta },t)$ at unseen parameter vectors $\tilde{\theta }$ and time t are obtained using the GP predictive mean defined in equation (25) of the electronic supplementary material.

### (b) Polynomial chaos expansion

PCE emulators are widely successful in multiple engineering applications [24,36] in their ability to quantify parameter importance and output uncertainty. The PCE approximates the true simulator, f(θ,t), by a truncated polynomial representation. The PCE polynomials are chosen to be orthogonal with respect to the prior distribution of the parameters. We assume uniform priors on all our parameters (after mapping them to the interval [−1,1] [36]) corresponding to Legendre polynomials. The polynomial coefficients, Z, are determined using a regression, ordinary least squares (OLS) approach [12]. The total number of PCE coefficients (per output) is J=((d+K)K), where d is the number of parameters and K is the polynomial order. Using F as defined in (3.1), the coefficients of the PCE are calculated as $Z={\left(\Psi^{⊤}\Psi \right)}^{-1}\Psi^{⊤}\text{F}$, where Ψ is the matrix of polynomials at each parameter value. The size of both the polynomial matrix Ψ and the coefficient matrix Z is dictated by the size of the solution space in F. Details regarding the PCE method and emulation training are relegated to the electronic supplementary material, in Section 5.

*PCE with Principal Component Analysis*: For q=5 principal components, the PCE requires $\Psi \in R^{J\times \left(5\times n\right)}$ and $\Psi \in R^{J\times 5}$. The assumption of independent outputs in the PCA representation parallels the assumption of independent PCE coefficients. We consider polynomial orders K=1,2,3,4,5,6. For the PCA representation, this only requires five sets of polynomial coefficients, which are computed by regressing on the PCA scores, that is, for the $k^{\text{th}}$ principal component score:


(4.5) $$c_{k}(\theta )=\sum_{j=0}^{J-1}z_{jk}\Psi_{j}(\theta ),$$

similar to the GP description in (4.3). PCE predictions map the parameter vector $\tilde{\theta }$, using the polynomial bases and coefficients defined in (4.5) to the PCA scores, which can then be transformed into the time domain using (4.2).

*PCE for time series*: When using the m=32 time-series output from the model, the PCE method assumes that the polynomial coefficients at each t are independent, and thus requires a polynomial matrix and coefficient matrix $\Psi \in R^{J\times \left(m\cdot n\right)}$ and $\Psi \in R^{J\times m}$, with n=100 or n=1000, as described earlier. PCE coefficients are determined using the OLS formulation. We consider PCEs with polynomial orders K=1,2,3,4,5,6, corresponding to J=5,15,35,70,126,210 PCE coefficients for d=4 parameters, and J=6,21,56,126,252,464 PCE coefficients for d=5 parameters. The polynomial coefficients are determined for each time point, and hence the coefficients themselves are time-dependent, that is,


(4.6) $$f(\theta ,t)=\sum_{j=0}^{J-1}z_{j}(t)\Psi_{j}(\theta ).$$

This is a typical approach to handling time-dependent outputs with PCE [12,24], and is available in software packages [27]. Unseen parameter vectors $\tilde{\theta }$ are mapped to the [−1,1] interval, and provide a time-dependent response for each polynomial basis-coefficient pair defined in (4.5).

## 5. Simulation set-up and performance assessment

### (a) Sensitivity analysis

We first perform a global sensitivity analysis using variance-based Sobol’ indices (through the PCE coefficients, z [12]) to identify influential parameters by the first- and total-order generalized Sobol’ indices [25], $S_{i}$ and $S_{T_{i}}$, respectively. These indices measure parameter importance, and we use the difference in magnitude to quantify parameter interactions that may cause identifiability issues [12]. Details regarding methodology, analysis and sensitivity results are in the electronic supplementary material, Section 6.2. This results in the reduced parameter subset for the WK and ST models:


(5.1) $$\theta_{\text{WK}}^{\text{infer}}=\left\{k_{1},k_{2},r_{\text{p}},r_{\text{d}},c_{\text{T}}\right\};\;\theta_{\text{ST}}^{\text{infer}}=\left\{k_{1},k_{2},\alpha ,\ell_{\text{rr}}\right\}.$$

### (b) Error metrics

We examine the effects of different assumptions on the training, validation, calibration and prediction for the four emulator options. After training the emulators with different training sizes (n=100 and n=1000), we perform validation to select the optimal hyperparameters (i.e. jitter value and kernel type for GPs and polynomial order for PCEs). We then fix these hyperparameters, and use them for testing data; see figure 1 for a summary. We investigate the effect of including two distinct representations of the model output (m=32 time points versus q=5 principal components) and the assumption of the emulator (GPs versus PCEs), as well as the effect of using training sizes of 100 and 1000. We assess the accuracy and robustness of each emulation approach for forward and inverse problems on testing data, and below we define the *error metrics* used (figure 1).

*Forward problem*: For both output representations (multivariate and PCA), we quantify the emulation error in *full output space* (i.e. m=32 points time series) by calculating the mean square error (MSE) between the simulator and the emulator at the validation or testing parameters through


(5.2) $$\text{MSE}(\theta_{j}^{\text{valid/test}})=\frac{1}{m}\sum_{i=1}^{m}(f(\theta_{j}^{\text{valid/test}},t_{i})-M(\theta_{j}^{\text{valid/test}},t_{i}){)}^{2},$$

for each validation or testing parameter $\theta_{j}^{\text{valid/test}},j=1,2,\ldots 100$ using the emulator, either $M^{\text{PCE}}(\cdot )$ or $M^{\text{GP}}(\cdot )$. For the PCA representation of the output, we transform the emulator predictions of the PCA scores back to the original time-dependent output space, see §4a,b for details.

*Inverse problem*: For the optimization of model parameters, θ, we implement a gradient-based optimization algorithm (sequential quadratic programming [37]) using the emulator. To reduce the risk of entrapment in local optima, we use 20 initial parameter values to minimize the MSE between the test data and emulator predictions. The inferred parameter vector, $\hat{\theta }$, obtained from the optimization is used for simulator prediction, $f(\hat{\theta },\text{t})$, to compute the simulator-based MSE, given by the first equation in (5.3). The relative square error (RSE) (middle equation in (5.3)) and absolute relative error (ARE) (last equation in (5.3)) measure the relative and absolute relative errors for each parameter:


(5.3) $$\text{MSE}({\hat{\theta }}_{j})=\frac{1}{m}\sum_{i=1}^{m}(f(\theta_{j}^{\text{test}},t_{i})-f({\hat{\theta }}_{j},t_{i}){)}^{2};\;\text{RSE}({\hat{\theta }}_{j})=\sum_{l=1}^{d}(\frac{\theta_{j,l}^{\text{test}}-{\hat{\theta }}_{j,l}}{\theta_{j,l}^{\text{test}}}{)}^{2};\;\text{ARE}({\hat{\theta }}_{l})=|\frac{\theta_{l}^{\text{test}}-{\hat{\theta }}_{l}}{\theta_{l}^{\text{test}}}|,$$

where $\text{MSE}({\hat{\theta }}_{j})$ and $\text{RSE}({\hat{\theta }}_{j})$ are computed for $j^{\text{th}}$ test (out-of-sample) dataset, and d in the expression for RSE denotes the cardinality of the parameter vector θ (d=5 for the WK model and d=4 for the ST model (5.1)).

### (c) Software

All simulations were run in Matlab (Mathworks, Natick, MA, USA). The GPstuff toolbox [35] was used to construct the GP models. A modification of the toolbox was needed for the GP time approach to accommodate the Kronecker product approximation. The UQlab toolbox [27] was used for PCE construction and evaluation.

## 6. Results

### (a) Hyperparameter selection

We train both GP and PCE emulators on the models using the parameters defined in (5.1). We inspect errors in output space based on validation data, computed with (5.2) for all simulation scenarios. The validation study allows us to select the optimal hyperparameters that give the *lowest median MSE* (5.2) over all validation datasets.

*GP jitter effect*: Our investigation (as supported through figures S2 and S3 in the electronic supplementary material) reveals that in general there is great overlap between MSE distributions of different jitters. The largest jitter value ($10^{-4}$) tends to produce the largest errors for the GP.time approach, but this does not hold for GP.PCA. Generally, GP.PCA seems to be less affected by the jitter value than GP.time. There is no universal ‘best’ jitter value, and for the analysis that follows next, we use the ‘best’ kernel-specific jitter value.

**Figure 2.**
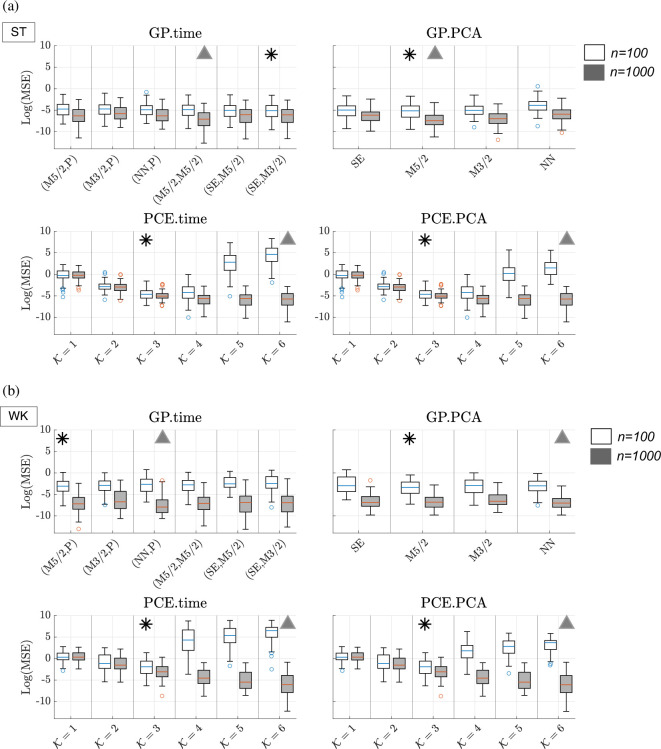
Emulation results based on validation data to select optimal hyperparameters (GP kernel type and PCE polynomial order). We show results from GPs (top row of panels (*a*) and (*b*) and PCEs (bottom row of panels (*a*) and (*b*) on the ST (panel (*a*)) and WK (panel (*b*)) boundary conditions with two output representations (time series -- first column -- and reduced PCA output -- second column --). GP kernels are: squared exponential (SE), Matèrn 3/2 (M3/2), Matèrn 5/2 (M5/2), neural network (NN) and periodic (P), for example, for GP.time, (M5/2, P) means Matèrn 5/2 kernel for biophysical parameters and periodic kernel for time input. We consider polynomial orders, K from 1 to 6. Boxplots show the log MSE (equation 5.2) from all 100 validation datasets. We consider n=100 (white-filled boxplots) and n=1000 training points (grey-shaded boxplots). We mark (black asterisks for n=100 and grey triangles for n=1000) the `best’ emulator hyperparameters for the *lowest median MSE* over the validation data.

In figure 2, we plot the distribution of log(MSE) (equation 5.2) over all 100 validation datasets for the ST (panel a) and WK model (panel b) under all simulation scenarios considered. We single out the ‘best’ kernel (GPs) for every simulation scenario, which we mark with a black asterisk (for n=100 training points) and grey triangle (for n=1000). The median values and interquartile range for each GP kernel, PCE order, sample size and formulation of the output signals are provided in electronic supplementary material, Tables S1–S4.

*GP kernel effect*: Generally, there is great overlap between MSE distributions of different kernels. There is no universal ‘best’ kernel. For the ST model (figure 2(a)), for the PCA approach (top row, right column), the Matèrn 5/2 (M5/2) kernel is best for both n=100 (white-filled boxplots) and n=1000 training points (grey-shaded boxplots). For the time approach, the combination of squared exponential (SE) kernel for parameters and Matèrn 3/2 (M3/2) for time input, (SE, M3/2), is best for n=100 training points, while the combination of M5/2 for parameters and for time input, (M5/2, M5/2), is best for n=1000 training points. For the WK model (figure 2(b)), for both time and PCA approaches (top row), for n=100 training points, M5/2 is best for the biophysical parameters (and periodic kernel for time input, (M5/2, P)), and for n=1000 training points, the NN kernel is best for parameters (and periodic kernel for time, (NN, P)).

*PCE polynomial order effect*: The polynomial order, K, affects emulator accuracy for both n=100 and n=1000 training size. For both the ST and WK model (figure 2(a,b), respectively), polynomial order K=3 provides the most accurate emulator for n=100 training points—marked with a black asterisk—for both time and PCA approaches (bottom row). The best emulator for n=1000—marked with a grey triangle—is provided by K=6. Polynomial orders K=4,5,6 are substantially worse with n=100 training points, a result of the overdetermined coefficient matrix. By contrast, the PCE accuracy steadily increases with K for n=1000 training points.

### (b) Out-of-sample evaluation

After selecting the optimal hyperparameters, we utilize the emulators for forward and inverse problems on test data. This procedure could be followed in the clinical practice prior to clinical data acquisition.

#### (i) Forward problem: direct emulation

**Figure 3.**
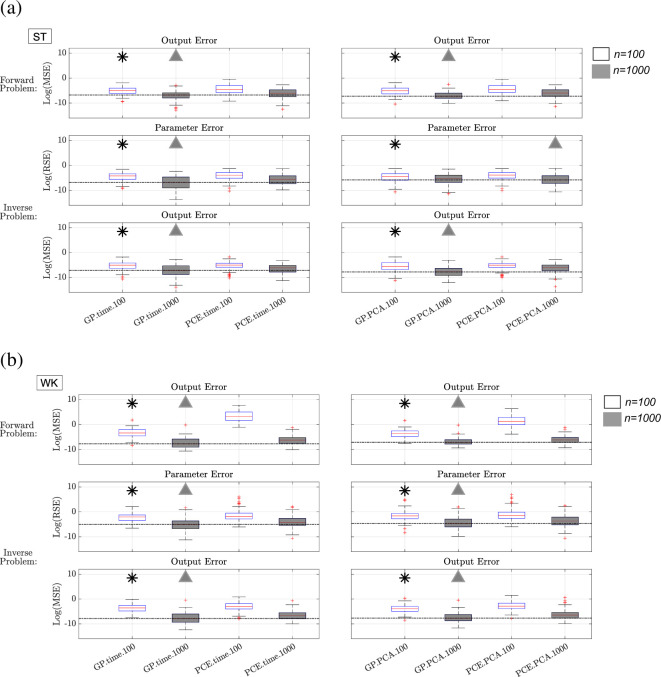
Results from forward and inverse problems using GP and PCE emulators for the ST (panel (*a*)) and WK (panel (*b*)) boundary conditions. Results are shown for both the time-series data representation (left column) and the PCA representation of the time-series data (right column). For the forward problem, we show boxplots based on the log of the MSE from all 100 test datasets, computed using equation (5.2). For the inverse problem, we show boxplots based on the log of the RSE in parameter space, and MSE in output space from all 100 test datasets, calculated using the first and second equations in (5.3). The black asterisks mark the superior emulator with n=100 training points, while the grey triangles mark the best emulator with n=1000 training points. The dotted lines in each plot correspond to the median of the best emulator for each metric.

We inspect prediction (emulation) errors in output space, that is, log⁡(MSE) (equation 5.2) on test data, which we show in figure 3 (top row in panels (a) and (b)). We mark the best emulator with symbols (black asterisks for n=100 training points and grey triangles for n=1000 training points). To ease a visual direct comparison with the other emulators, we draw a horizontal (reference) line corresponding to the median MSE across all 100 test datasets of the best emulator.

*Training size and data representation*: For both GP and PCE emulators, the errors are systematically lower for the largest training size of n=1000 (grey-shaded boxplots) compared with n=100 (white-filled boxplots), and this effect is more pronounced for the WK model than the ST model. A larger training size ensures a denser coverage of the parameter space, which increases the emulator predictive accuracy. While for PCE there is a clear reduction in accuracy for the n=100 training size as K increases, attributed to an overdetermined system for the PCE coefficients, larger training sizes enable higher order PCE models to be accurately trained. For both GP and PCE, there is no strong evidence of either (time series, PCA) approach being better than the other, as there is great overlap in error distributions.

*GP versus PCE*: Here, we compare GP.PCA with PCE.PCA, and GP.time with PCE.time. We observe that the PCE errors tend to be slightly higher than the GP errors. GP.time and GP.PCA are consistently at least one order of magnitude smaller than the PCE.time and PCE.PCA models. This is clear for the WK model, where GP.time.100 and GP.PCA.100 are of the order of $10^{-2}$, whereas PCE.time.100 and PCE.PCA.100 are $10^{1}$ and $10^{0}$, respectively. This finding suggests that GP emulators have a slightly larger predictive accuracy than PCE emulators given the fixed emulator design constructed based on a Sobol sequence.

We conclude that with respect to the out-of-sample predictive accuracy in output space (forward problem), the two best methods are GP.time and GP.PCA with 1000 training points.

#### (ii) Inverse problem: parameter estimation

We inspect errors after performing parameter estimation on test data, as described in §5. In figure 3, we display the errors in both output (middle row in panels (a) and (b)) and parameter space (bottom row in panels (a) and (b)), obtained with the first and second equations in (5.3).

*Training size and data representation*: As with the forward problem, emulators with the larger training size of 1000 systematically produce more accurate parameter estimates and corresponding predicted outputs than the 100 training points counterparts. This effect is slightly more pronounced for the WK model than the ST model. When comparing GP.PCA with GP.time, and PCE.PCA with PCE.time in parameter and output space, we notice great overlap in error distributions. Hence, all emulators exhibit similar performance, which aligns with findings from the forward problem.

*GP versus PCE*: When comparing GP.PCA with PCE.PCA and GP.time with PCE.time, we observe that, as with the forward problem, GPs tend to have a slight advantage over PCEs. The inverse problem RSE values (5.3) for n=100 are of the order of $10^{-2}$ and $10^{-1}$ for ST and WK models, respectively. The RSE for GPs and PCEs with n=1000 are of the order of $10^{-3}$ for the ST model. The GPs achieve a notable improvement in RSE for n=1000 in the WK model (GP RSEs of $10^{-1}$ and $10^{-3}$ for n=100 and n=1000, PCE RSEs $10^{-1}$ and $10^{-2}$ for n=100 and n=1000). The ST RSE is slightly smaller for PCE.PCA.1000 ($3.0\times 10^{-3}$ for PCE versus $4.7\times 10^{-3}$ for GPs); however, PCE.PCA.1000 has a wider distribution of AREs, and subsequently accrues a larger MSE in output space. These additional results can be found in the electronic supplementary material, Section 8.

Both GPs and PCEs record similar MSE errors (5.3) for both the ST and WK models ($10^{-3}$ and $10^{-2}$, respectively) for n=100. By contrast, GPs achieve a smaller MSE model for n=1000 in both the ST and WK models ($10^{-4}$) than the PCEs ($10^{-3}$).

**Figure 4.**
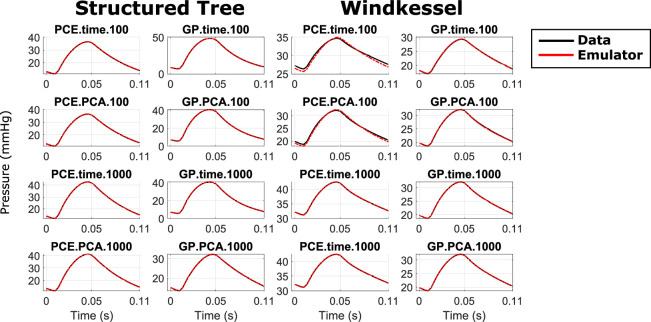
Optimum simulator solution using the model with the worst performance on the test data, for which the parameter optimization returned the largest error in parameter space, RSE (middle equation in (5.3)) out of all 100 test datasets. Data are shown in solid black lines while emulator-determined simulator predictions are shown as dash-dot red lines.

In summary, our finding is that GP.time and GP.PCA with 1000 training points are generally the two best methods by both output and input space metrics. These were the two best methods with respect to the forward problem too, implying consistency in results between the forward and inverse problems. While GPs outperform PCEs in nearly every metric, we note that the qualitative differences, especially in output space, are less obvious. We subsequently identified the largest RSE out of all 100 test datasets for each model after solving the inverse problem, and used these parameters to run the true simulator. The results from this are presented in figure 4. Note that, with the exception of PCE.time.100 in the WK model, the solutions are nearly identical visually, with indistinguishable differences between the prediction with the emulator-inferred parameter values and the true data. The difference between the data and the emulator is noticeably smaller than the expected error obtained from clinical measurements [20].

## 7. Discussion

Surrogate models are necessary to develop digital twins that not only simulate patient-specific dynamics but can also be calibrated to patient data in real time (e.g. within the time resolution of a single heartbeat). While the GP and PCE emulation strategies are not new in the field of engineering, their use in biological and medical applications is relatively novel and requires detailed investigations, as presented here. Moreover, we provide results suggesting that GPs slightly outperform PCEs, and find no difference in accuracy metrics when using PCA for dimension reduction.

### (a) Direct emulation

Numerous articles use GPs [13,38] and PCEs [10,36,39] to speed up expensive simulators, many for the calculation of sensitivity indices. Other studies [40] leverage information from lower-fidelity (but more computationally efficient) simulations to increase emulation accuracy. The multi-fidelity modelling approach reduces computational cost compared with using a single high-fidelity model with a limited number of simulations.

Regardless of purpose, investigating which emulators are most accurate is paramount, and testing the emulator predictions against out-of-sample ‘test’ data is a necessary step. Here, we first optimized emulator hyperparameters (kernel type and jitter term for GPs or polynomial order for PCEs) in a validation study, and then evaluated the emulator performance with fixed hyperparameters on test data. We investigated the effect of (i) representations of the model output (time-series output, or PCA representation) and two emulators (GPs or PCEs), and (ii) different training sizes.

We show that the choice of GP kernel and jitter does not have a drastic effect on emulator accuracy. However, the effect of the PCE polynomial order is significant (figure 2). In addition, our evaluation on test data revealed that the emulation accuracy is similar for the two types of output representations, multivariate output (time series) and PCA. We find that GPs outperform PCEs slightly, but consistently, for ST and WK models (figure 3). We found that the predictive accuracy is improved when a larger training size is used, due to a denser coverage of the parameter space (figure 3). Our conclusion is that with respect to emulation accuracy, the two best methods are GP.time and GP.PCA with the larger training size, and this applies to both fluid-dynamics models. We also note that emulating the model with ST boundary conditions was less sensitive to changes in sample size. We attribute this to both the smaller dimensionality of the problem (four-dimensional versus five-dimensional), as well as the relatively lower variance of the ST model as calculated in the electronic supplementary material, figure S6.

### (b) Parameter estimation using emulators

Most emulation studies have an end goal of parameter estimation and/or uncertainty quantification [6,11]. In our study, we focus on parameter estimation on test data using the emulators with optimal hyperparameters based on a validation study. This mimics clinical practice, in which the ‘best’ emulator is identified prior to new patient data becoming available and is immediately utilized for *real-time* patient-specific inference.

Our analysis focuses on a pulse–wave propagation model of the pulmonary circulation, which has been used in multiple prior studies [15,16,20,39]. The studies by Qureshi *et al*. [20] and Colebank *et al*. [14] calibrated their one-dimensional haemodynamics models with WK boundary conditions to measured haemodynamic data using the full simulator. These studies required a significant amount of computation time for the calibration step. For instance, Colebank *et al*. [14] reported that optimization took in the order of hours, whereas optimization in the present study using the emulator took less than a minute. Paun & Husmeier [11] used GPs as a surrogate for the log-likelihood for the fluids model presented here with WK boundary conditions. The authors showed a drastic increase in computation time and performance across multiple methods for Bayesian inference. In contrast to these prior studies, relatively few papers have performed parameter estimation using the ST boundary condition.

The inverse problem results in figure 3 illustrate that the inference accuracy in both output and input (parameter) space is similar between the two types of output representations, and that GPs tend to have a slight advantage over PCEs. The figure also shows that there is an improvement in both parameter estimates and, subsequently, fit in output space when using larger training sets. In summary, we have found that with respect to inference accuracy, generally the two best methods are GP.time and GP.PCA with the larger training size for both fluid-dynamics models, which aligns with emulation accuracy results.

### (c) Emulators for PCA versus time representation

Our study is one of the very few that comparatively evaluates the performance between PCA emulators and emulators for multivariate outputs, and to the best of our knowledge, it is the first study that compares GP.PCA with the particular GP.time strategy adopted here, that is, treating time as an additional input to the GP model. Additionally, the comparison between PCA and time series in a PCE emulation context is novel.

Our results indicate similar performance in output predictive accuracy and parameter inference accuracy between PCA and time-series emulators (figure 3) for both GPs and PCEs. It therefore appears that the information loss incurred from the finite truncation of the PCA decomposition of the simulator output is on a par with the information loss from the Kronecker product approximation of the GP.time approach, or the independence assumption between the time points for the PCE.time approach.

Both PCA and time approaches are comparable in computation time provided the training of independent emulators (e.g. for principal component scores) is done in parallel, and the same holds for prediction. For reference, training requires 1 min, prediction at one sample test point requires a few milliseconds, while the entire optimization takes approximately 1−2 minutes for both GPs and PCEs.

### (d) Gaussian processes versus polynomial chaos expansions

Our findings indicate that GP emulators have a slightly higher predictive and inference accuracy than PCE emulators. As noted earlier, both emulators have similar orders of magnitude with the exception of the WK model and 100 training points, where GPs outperform PCEs by one to two orders of magnitude in output error on the test set. This finding holds for the fixed space-filling design constructed based on a Sobol sequence. We have used the Sobol sequence as a standard textbook design for convenience, as this was sufficient for the purpose of our study. Sobol sequences have their limitations in terms of idiosyncratic clustering of design points, but that only comes to the fore in higher dimensions than studied in our paper (e.g. figs 7.11 and 7.12 in [22]). Optimal sequential design can potentially lead to design improvements. However, this requires more complex modelling, which is beyond the remit of the current work, while simpler ‘intuitive’ approaches, based, for example, on mutual information, can lead to suboptimal configurations with an accumulation of design points at the margins of the compact design space; see, for example, Chapter 6 in [41]. The interested reader is referred to [22] (Chapter 7) and [42] for a review on optimal experimental designs for emulators of computerized simulation models. Some studies have investigated the effect of changing the experimental design on sensitivity analysis and model calibration [6,43]. Investigating the effect of changing the experimental design is beyond the remit of this work.

We also note here that there is a direct (and fair) comparison between GP.PCA with PCE.PCA, both of which capture output correlations at different time points through PCA decomposition. By contrast, the comparison of GP.time with PCE.time is confounded by the way in which the correlation between time points is captured, and comparative results should be interpreted with caution. The GP.time approach captures the correlation between time points by the inclusion of time as an emulator input with its own covariance function, whereas PCE.time considers independent coefficients (using the same polynomial basis functions) for each time point through time-dependent coefficients (4.5). Approaches that explicitly capture the correlation between polynomial coefficients at different time points are an active area of research [26,44]. In particular, we believe explicitly accounting for these correlations through varying coefficient models [45] is of interest moving forward.

While the current study has considered GPs and PCEs as two separate emulation methods, the two could be married in a polynomial chaos-based GP approach, in which PCE describes the mean function of the GP [46,47]. In this approach, adopted by several studies in the literature [46,47], the PCE models the global behaviour, while the GP captures the local variability of the simulator output. For example, the study in [47] showed that while this approach comes at increased computational costs, it tends to perform at least as well as, or in certain situations better than GP or PCE emulators on their own, rendering this approach worthy of future investigations.

We also emphasize that, while PCEs are often used as a surrogate to speed up computation for global sensitivity analysis, they are less often used for additional forward solutions and in parameter estimation problems. Though GPs are superior by our reported metrics, PCEs are reasonably accurate in both forward and inverse problems. Hence, we recommend that those using PCEs for sensitivity analysis consider using their PCE surrogate to overcome computation time when conducting parameter estimation.

### (e) Limitations and future work

In the current study, we have considered an idealized case of noise-free, simulator-generated data for inference. To focus on emulation error in itself and eliminate potentially confounding factors we have deliberately ignored model discrepancy, as well as noise. Additionally, using simulated data has allowed us to compare parameter estimates to ‘ground-truth’ (data-generating) parameter values. In future simulation studies, we will use noisy data, representative of clinical patient data. The noise model needs to be physiologically realistic (not Gaussian independent and identically distributed), which is not readily available ‘off the shelf’, and so this is beyond the remit of the present work. We will then perform inverse uncertainty quantification in a Bayesian framework, similar to other recent studies [3], and investigate how the posterior densities compare. Any Bayesian analysis performed on patient data will capture the uncertainty due to (i) data measurement error, and (ii) model discrepancy due to model simplifications [16]. Model mismatch is particularly important during model calibration against real patient data, as failing to account for the mismatch will lead to biased parameter estimates and predictions, and an uncertainty underestimation, as shown in several studies [16,48]. To incorporate model mismatch into the analysis, the Kennedy & O’Hagan approach [32] could be taken to jointly learn the model parameters and model mismatch term from data, while ideally incorporating system knowledge [48], or the problem could be formulated as a decoupled inference problem, by adopting methods such as Bayesian History Matching [49]. Given that addressing model mismatch is highly relevant for the clinical translation of cardiovascular modelling, incorporating it into our model calibration within an emulation framework will certainly be pursued in future studies when clinical data are made available. What is more, while beyond the remit of the present work, additional comparisons using gradient-informed emulators, such as gradient-enhanced Kriging [50], are warranted, especially when solving inverse problems through gradient-based methods.

Additionally, it will be interesting to extend the emulation method comparison and investigate whether our findings generalize to other biological systems besides the pulmonary blood circulation application considered here, such as the systemic blood circulation [2]. The systemic vasculature (e.g. the coronary circulation around the heart or the cerebrovascular in the brain) contribute to two major causes of death, heart attack and stroke. An extension of our analysis to these distinct vasculature with patient-specific data would provide evidence of whether a robust and efficient *digital twin* could be used for treatment planning and diagnostics in these common diseases. Moreover, we believe our methods should be tested against multi-component models, such as those with models of the heart [51] and venous system [39,51], which will likely be more challenging due to higher parameter dimensionality. These are necessary next steps as models and emulators are used in practice clinically.

## 8. Conclusions

Our study has investigated the use of emulation as a key enabler of real-time personalized healthcare in a computational modelling framework of the cardiovascular system. We have comparatively assessed several emulation strategies based on GPs and PCEs applied to two computational fluid-dynamics models of the pulmonary blood circulation. After reducing the parameter space of the models through global sensitivity analysis, we have created emulators of the time-series model output, and of a PCA-reduced representation of the original, multivariate output. We have assessed the emulators’ out-of-sample predictive accuracy, as well as their inference accuracy in inverse problems for parameter estimation tasks. We have found that GPs outperform slightly, but consistently, polynomial chaos expansions across every comparison, and that a similar performance is obtained for the emulators of multivariate output and reduced output.

## Data Availability

Data are available on Zenodo [52]. Supplementary material is available online [53].

## Footnotes

^1^
The GP predictive variance could be used to allow uncertainty quantification; however, this is beyond the remit of this work.

## References

[1] Niederer SA, Sacks MS, Girolami M, Willcox K. 2021 Scaling digital twins from the artisanal to the industrial. Nat. Comput. Sci. **1**, 313–320. (10.1038/s43588-021-00072-5) 38217216

[2] Yang S *et al*. 2024 Long-term prognostic implications of CT angiography-derived fractional flow reserve: Results from the DISCOVER-FLOW study. J. Cardiovasc. Comput. Tomogr. **18**, 251–258. (10.1016/j.jcct.2024.01.016) 38378313

[3] Laloy E, Jacques D. 2019 Emulation of CPU-demanding reactive transport models: a comparison of Gaussian processes, polynomial chaos expansion, and deep neural networks. Comput. Geosci. **23**, 1193–1215. (10.1007/s10596-019-09875-y)

[4] Pratola MT, Higdon DM. 2016 Bayesian additive regression tree calibration of complex high-dimensional computer models. Technometrics **58**, 166–179. (10.1080/00401706.2015.1049749)

[5] Zhe S, Xing W, M.Kirby R. 2019 Scalable high-order Gaussian process regression (eds K Chaudhuri, M Sugiyama). In Proc. 22nd Int. Conf. Artificial Intelligence and Statistics, 16–18 April, vol. 89, pp. 2611–2620, PMLR.

[6] Owen NE, Challenor P, Menon PP, Bennani S. 2017 Comparison of surrogate-based uncertainty quantification methods for computationally expensive simulators. SIAM/ASA J. Uncertainty Quantif. **5**, 403–435. (10.1137/15M1046812)

[7] Sun X, Pan X, Choi JI. 2021 Non-intrusive framework of reduced-order modeling based on proper orthogonal decomposition and polynomial chaos expansion. J. Comput. Appl. Math. **390**, 113372. (10.1016/j.cam.2020.113372)

[8] Arzani A, Dawson STM. 2021 Data-driven cardiovascular flow modelling: examples and opportunities. J. R. Soc. Interface **18**, 20200802. (10.1098/rsif.2020.0802) 33561376 PMC8086862

[9] Perez-Raya I, Fathi MF, Baghaie A, Sacho R, D’Souza RM. 2021 Modeling and reducing the effect of geometric uncertainties in intracranial aneurysms with polynomial chaos expansion, data decomposition, and 4D-flow MRI. Cardiovasc. Eng. Technol. **12**, 127–143. (10.1007/s13239-020-00511-w) 33415699

[10] Donders WP, Huberts W, van de Vosse FN, Delhaas T. 2015 Personalization of models with many model parameters: an efficient sensitivity analysis approach. Int. J. Numer. Methods Biomed. Eng. **31**, 2727. (10.1002/cnm.2727) 26017545

[11] Paun LM, Husmeier D. 2022 Emulation-accelerated Hamiltonian Monte Carlo algorithms for parameter estimation and uncertainty quantification in differential equation models. Stat. Comput. **32**, 25. (10.1007/s11222-021-10060-4)35310544

[12] Eck VG, Donders WP, Sturdy J, Feinberg J, Delhaas T, Hellevik LR, Huberts W. 2016 A guide to uncertainty quantification and sensitivity analysis for cardiovascular applications. Int. J. Numer. Method. Biomed. Eng. **32**, 8. (10.1002/cnm.2755) 26475178

[13] Melis A, Clayton RH, Marzo A. 2017 Bayesian sensitivity analysis of a 1D vascular model with Gaussian process emulators. Int. J. Numer. Method. Biomed. Eng. **33**, 1–11. (10.1002/cnm.2882) 28337862

[14] Colebank MJ, Umar Qureshi M, Olufsen MS. 2021 Sensitivity analysis and uncertainty quantification of 1‐D models of pulmonary hemodynamics in mice under control and hypertensive conditions. Int. J. Numer. Methods Biomed. Eng. **37**. (10.1002/cnm.3242) 31355521

[15] Chambers MJ, Colebank MJ, Qureshi MU, Clipp R, Olufsen MS. 2020 Structural and hemodynamic properties of murine pulmonary arterial networks under hypoxia-induced pulmonary hypertension. Proc. Inst. Mech. Eng. Part H **234**, 1312–1329. (10.1177/0954411920944110) 32720558

[16] Paun LM, Colebank MJ, Olufsen MS, Hill NA, Husmeier D. 2020 Assessing model mismatch and model selection in a Bayesian uncertainty quantification analysis of a fluid-dynamics model of pulmonary blood circulation. J. R. Soc. Interface **17**, 20200886. (10.1098/rsif.2020.0886) 33353505 PMC7811590

[17] van de Vosse FN, Stergiopulos N. 2011 Pulse wave propagation in the arterial tree. Annu. Rev. Fluid Mech. **43**, 467–499. (10.1146/annurev-fluid-122109-160730)

[18] Olufsen MS, Peskin CS, Kim WY, Pedersen EM, Nadim A, Larsen J. 2000 Numerical simulation and experimental validation of blood flow in arteries with structured-tree outflow conditions. Ann. Biomed. Eng. **28**, 1281–1299. (10.1114/1.1326031) 11212947

[19] Westerhof N, Lankhaar JW, Westerhof BE. 2009 The arterial windkessel. Med. Biol. Eng. Comput. **47**, 131–141. (10.1007/s11517-008-0359-2) 18543011

[20] Qureshi MU, Colebank MJ, Paun LM, Ellwein Fix L, Chesler N, Haider MA, Hill NA, Husmeier D, Olufsen MS. 2019 Hemodynamic assessment of pulmonary hypertension in mice: a model-based analysis of the disease mechanism. Biomech. Model. Mechanobiol. **18**, 219–243. (10.1007/s10237-018-1078-8) 30284059

[21] Bratley P, Fox BL. 1988 Algorithm 659: Implementing Sobol’s quasirandom sequence generator. ACM Trans. Math. Softw. **14**, 88–100. (10.1145/42288.214372)

[22] McClarren RG. 2018 Uncertainty quantification and predictive computational science: a foundation for physical scientists and engineers, 1st edn. Cham, Switzerland: Springer. (10.1007/978-3-319-99525-0_1)

[23] Roberts S, Osborne M, Ebden M, Reece S, Gibson N, Aigrain S. 2013 Gaussian processes for time-series modelling. Philos. Trans. A. Math. Phys. Eng. Sci. **371**, 20110550. (10.1098/rsta.2011.0550) 23277607

[24] Eck VG, Sturdy J, Hellevik LR. 2017 Effects of arterial wall models and measurement uncertainties on cardiovascular model predictions. J. Biomech. **50**, 188–194. (10.1016/j.jbiomech.2016.11.042) 27890534

[25] Alexanderian A, Gremaud PA, Smith RC. 2020 Variance-based sensitivity analysis for time-dependent processes. Reliab. Eng. Syst. Saf. **196**, 106722. (10.1016/j.ress.2019.106722)

[26] Gerritsma M, van der Steen JB, Vos P, Karniadakis G. 2010 Time-dependent generalized polynomial chaos. J. Comput. Phys. **229**, 8333–8363. (10.1016/j.jcp.2010.07.020)

[27] Marelli S, Sudret B. 2014 UQLab: a framework for uncertainty quantification in Matlab. In Second International Conference on Vulnerability and Risk Analysis and Management (ICVRAM) and the Sixth International Symposium on Uncertainty, Modeling, and Analysis (ISUMA), Liverpool, UK, pp. 2554–2563. Reston, VA. (10.1061/9780784413609.257). http://ascelibrary.org/doi/book/10.1061/9780784413609.

[28] Higdon D, Gattiker J, Williams B, Rightley M. 2008 Computer model calibration using high-dimensional output. J. Am. Stat. Assoc. **103**, 570–583. (10.1198/016214507000000888)

[29] Rasmussen CE, Williams CKI. 2005 Gaussian processes for machine learning (adaptive computationand machine learning). Cambridge, MA: MIT Press.

[30] Conti S, O’Hagan A. 2010 Bayesian emulation of complex multi-output and dynamic computer models. J. Stat. Plan. Inference **140**, 640–651. (10.1016/j.jspi.2009.08.006)

[31] Conti S, Gosling JP, Oakley JE, O’Hagan A. 2009 Gaussian process emulation of dynamic computer codes. Biometrika **96**, 663–676. (10.1093/biomet/asp028)

[32] Kennedy MC, O’Hagan A. 2001 Bayesian calibration of computer models. J. R. Stat. Soc. Ser. B. **63**, 425–464. (10.1111/1467-9868.00294)

[33] Matheron G. 1963 Principles of geostatistics. Econ. Geol. **58**, 1246–1266. (10.2113/gsecongeo.58.8.1246)

[34] Andrianakis I, Challenor PG. 2012 The effect of the nugget on Gaussian process emulators of computer models. Comput. Stat. Data Anal. **56**, 4215–4228. (10.1016/j.csda.2012.04.020)

[35] Vanhatalo J, Riihimäki J, Hartikainen J, Jylänki P, Tolvanen V, Vehtari A. 2013 GPstuff: Bayesian modeling with Gaussian processes. J. Mach. Learn. Res **14**, 1175–1179. (10.5555/2567709.2502617)

[36] Huberts W, Donders WP, Delhaas T, van de Vosse FN. 2014 Applicability of the polynomial chaos expansion method for personalization of a cardiovascular pulse wave propagation model. Int. J. Numer. Methods Biomed. Eng. **30**, 1679–1704. (10.1002/cnm.2695) 25377937

[37] Boggs PT, Tolle JW. 2000 Sequential quadratic programming for large-scale nonlinear optimization. J. Comput. Appl. Math. **124**, 123–137. (10.1016/s0377-0427(00)00429-5)

[38] Tsokanas N, Pastorino R, Stojadinović B. 2021 A comparison of surrogate modeling techniques for global sensitivity analysis in hybrid simulation. M.A.K.E. **4**, 1–21. (10.3390/make4010001)

[39] Colebank MJ, Chesler NC. 2024 Efficient uncertainty quantification in a spatially multiscale model of pulmonary arterial and venous hemodynamics. Biomech. Model. Mechanobiol. **23**, 1909–1931. (10.1007/s10237-024-01875-x) 39073691 PMC11554845

[40] Ji Y, Yuchi HS, Soeder D, Paquet JF, Bass SA, Joseph VR, Wu CFJ, Mak S. 2024 Conglomerate multi-fidelity gaussian process modeling, with application to heavy-ion collisions. SIAM/ASA J. Uncertainty Quantif. **12**, 473–502. (10.1137/22M1525004)

[41] Gramacy RB. 2020 Surrogates: Gaussian process modeling, design and optimization for the applied sciences, pp. 143–221. Boca Raton, FL: Chapman and Hall/CRC. (10.1201/9780367815493-5)

[42] Challenor P. 2013 Experimental design for the validation of kriging metamodels in computer experiments. J. Simul. **7**, 290–296. (10.1057/jos.2013.17)

[43] Renardy M, Joslyn LR, Millar JA, Kirschner DE. 2021 To Sobol or not to Sobol? The effects of sampling schemes in systems biology applications. Math. Biosci. **337**, 108593. (10.1016/j.mbs.2021.108593) 33865847 PMC8184610

[44] Luchtenburg DM, Brunton SL, Rowley CW. 2014 Long-time uncertainty propagation using generalized polynomial chaos and flow map composition. J. Comput. Phys. **274**, 783–802. (10.1016/j.jcp.2014.06.029)

[45] Fan J, Zhang W. 2008 Statistical methods with varying coefficient models. Stat. Its Interface **1**, 179–195. (10.4310/sii.2008.v1.n1.a15) PMC2575822 18978950

[46] Jia B, Xin M. 2021 Active sampling based polynomial-chaos–kriging model for orbital uncertainty propagation. J. Guid. Control. Dyn. **44**, 905–922. (10.2514/1.G005130)

[47] Schobi R, Sudret B, Wiart J. 2015 Polynomial-chaos-based kriging. Int. J. Uncertain. Quantif. **5**, 171–193. (10.1615/Int.J.UncertaintyQuantification.2015012467)

[48] Brynjarsdóttir J, OʼHagan A. 2014 Learning about physical parameters: the importance of model discrepancy. Inv. Probl. **30**, 114007. (10.1088/0266-5611/30/11/114007)

[49] Gardner P, Rogers TJ, Lord C, Barthorpe RJ. 2021 Learning model discrepancy: A Gaussian process and sampling-based approach. Mech. Syst. Signal Process. **152**, 107381. (10.1016/j.ymssp.2020.107381)

[50] Ulaganathan S, Couckuyt I, Ferranti F, Laermans E, Dhaene T. 2015 Performance study of multi-fidelity gradient enhanced kriging. Struct. Multidiscip. Optim. **51**, 1017–1033. (10.1007/s00158-014-1192-x)

[51] Mynard JP, Smolich JJ. 2015 One-dimensional haemodynamic modeling and wave dynamics in the entire adult circulation. Ann. Biomed. Eng. **43**, 1443–1460. (10.1007/s10439-015-1313-8) 25832485

[52] Paun LM. 2024 LMihaelaPaun/Emulation_GP_PCE: Matlab Code (v1.1). Zenodo. (10.5281/zenodo.13961401)

[53] Paun M, Colebank MJ, Husmeier D. 2025 Supplementary material from: A comparison of Gaussian processes and polynomial chaos emulators in the context of haemodynamic pulse-wave propagation modelling. Figshare (10.6084/m9.figshare.c.7658929) PMC11904621 40078149

